# Castleman disease of the hyaline vascular variant transforming to POEMS syndrome as endpoint: a case report

**DOI:** 10.1186/s12883-018-1172-7

**Published:** 2018-10-09

**Authors:** Yijun Ge, Qian Da, Ying Dai

**Affiliations:** 1 the sleep disease center, the affiliated Chaohu Hospital of Anhui Medical University, Chaohu, China; 2 Pathology Department, the affiliated Ruijin Hospital of Shanghai Jiaotong University, Shanghai, China; 3 Oncology Department, the 1st Affiliated Hospital of Anhui Medical University, Jixi Road 218, Hefei, 230022 China

**Keywords:** POEMS syndrome, Castleman disease, Polyneuropathy

## Abstract

**Background:**

POEMS syndrome is a rare neoplastic syndrome reflected by plasma cell disorder. It is composed by polyneuropathy, organomegaly, endocrinopathy, monoclonal protein, and skin changes. It is also reported to associate with Castleman disease. The early identification and treatment are pivotal to reduce the morbidity and mortality.

**Case presentation:**

Here we report a 66-year-old man with treated Castleman disease developing with sequential presence of endocrinopathy polyneuropathy, skin changes, organomegaly and extravascular volume overload within 18 years, which was finally confirmed as POEMS syndrome by positive monoclonal protein. He was thereafter successfully treated with prednisone and azathioprine as primary therapy and thalidomide as maintenance therapy.

**Conclusion:**

The diagnosis of POEMS is based on a cluster of disorder involved in varied organs. We report a rare case that triggers the need to consider POEMS syndrome diagnosis for patients carrying Castleman disease and polyneuropathy.

## Background

Castleman disease (CD) is a rare disorder known as angiofollicular lymph node hyperplasia, which occurs in around 11–30% of patients with POEMS syndrome (Crow-Fukase syndrome) [1]. POEMS syndrome compromises polyneuropathy, organomegaly, endocrinopathy, monoclonal protein, and skin changes [2]. Endocrinopathy is the core component of the syndrome, reflected by hypogonadism, thyroid abnormalities, glucose metabolism abnormalities and adrenal insufficiency. Due to the unclear pathogenesis of the syndrome, risk stratification mainly relies on clinical phenotype instead of molecular marker and the extent of the plasma cell disorder is prognostic to determine therapeutic options [3].

## Case presentation

**Fig. 1:**
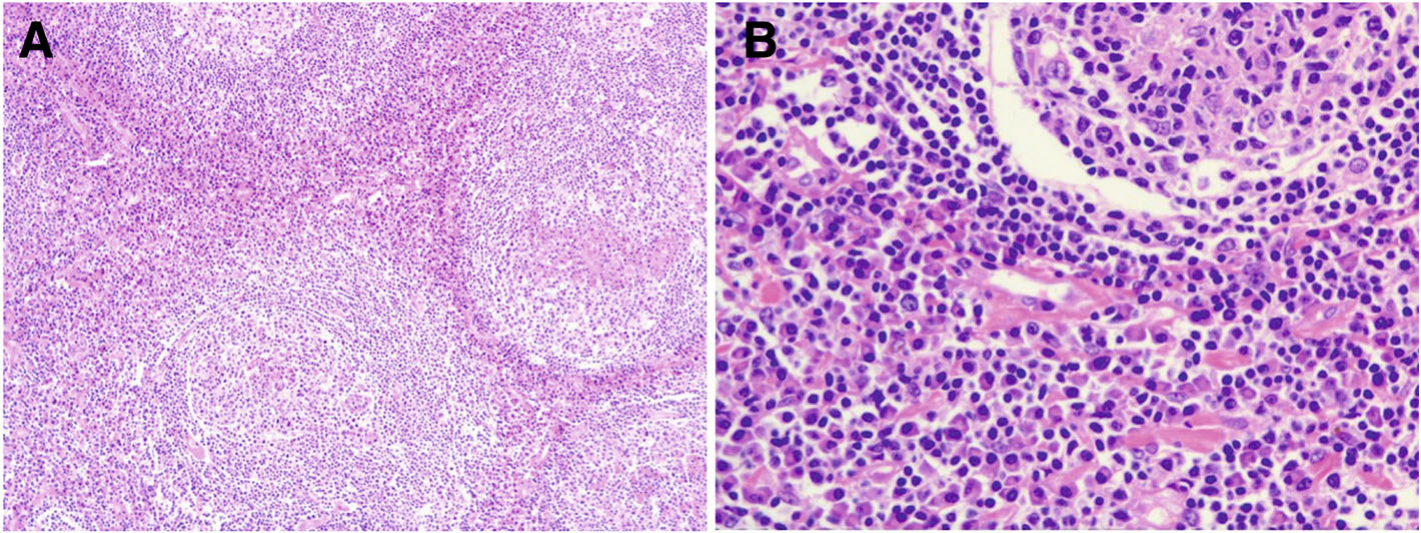
Pathology of the biopsied lymph node showing features of Castleman disease of hyaline vascular variant. B-cell follicle with concentric layers of lymphocytes expanding to the mantel zone, known as “onion skin”, hyalinized germinal center with increased density of blood vessels. **a** original magnification× 100; **b** original magnification× 400

A 66-year-old male presented with the left cervical and submaxillary lymph node enlargement with size of 30 mm × 30 mm. The following lymph node biopsy was completed and the pathological evaluation showed reactive lymphoid hyperplasia characterized by vascular, atrophic germinal lesions with surrounding concentric “onion skin” layers of lymphocytes (Fig. 1), defined as the hyaline vascular variant of CD. He denied weight loss, lymphadenopathy, tuberculosis, diabetes mellitus and hypertension. He gave no history of bone pain and drug abuse. The bone marrow (BM) cytology indicated no clonal plasma cell infiltration. Thereafter the patient was treated with irradiation dose of 30 Gy in 10 fractions with clinical response. The clinical evaluation remained stable in annual follow-up visits. In the 4th visit, the patient was diagnosed in an outside hospital with 2-diabetic mellitus on basis of increased blood glucose, C peptide release test and oral glucose tolerance test (OGTT). The blood glucose was well-controlled by the hypoglycemic and diet therapy for three years when he presented to the previous hospital with progressive hand-foot numbness spreading from the proximal extremes to distal ends. The electromyography test confirmed the hampered nerve conduction velocity of bilateral ulnar, median and peroneal nerves. The cerebrospinal fluid (CSF) biochemistry from lumbar puncture indicated the slightly elevated protein (1.1 g/L, normal, 0.1~ 0.4 g/L) with normal cell count. The blood monoclonal protein level and bone marrow cytology was normal. Based on the resultant diagnosis of chronic inflammatory demyelinating polyneuropathy (CIDP), the patient was treated with daily intravenous pulse methylprednisolone therapy of 1.0 g for three consecutive days. Due to the minor clinical response, the diabetic peripheral neuropathy was suspected with following insulin injection and oral mecobalamin therapy. However, the manifestation of hand-foot numbness remained constant. One and half years after the first sign of numbness, the muscular dystrophy of palms was noticed with finger fine hypoactivity. In the next 2 years, the patient was admitted to the previous hospital with the progressive muscular atrophy, numbness of hands and myasthenia of limbs along with interphalangeal deformity. The CSF test showed slightly elevated protein concentration (1.6 g/L) with normal pressure, cell count and the level of glucose and chloride. Other laboratory results were normal. The electromyography test was performed with severe neuro-electrophysiological damage in bilateral median, ulnar, peroneal and tibial nerves. Despite the intensive glucose control and oral mecobalamin therapy for the next seven years, the symptom of limb numbness and trembling was significantly worsened. In parallel, the skin pigmentation with increasing body hair and skin elasticity loss was also noted. He gave the history of insomnia for seven years but denied smoking and alcohol abuse history. Insulin and oral anti-diabetic drugs was thereafter withdrawn at the normal level of HbAlc and blood glucose.

**Fig. 2:**
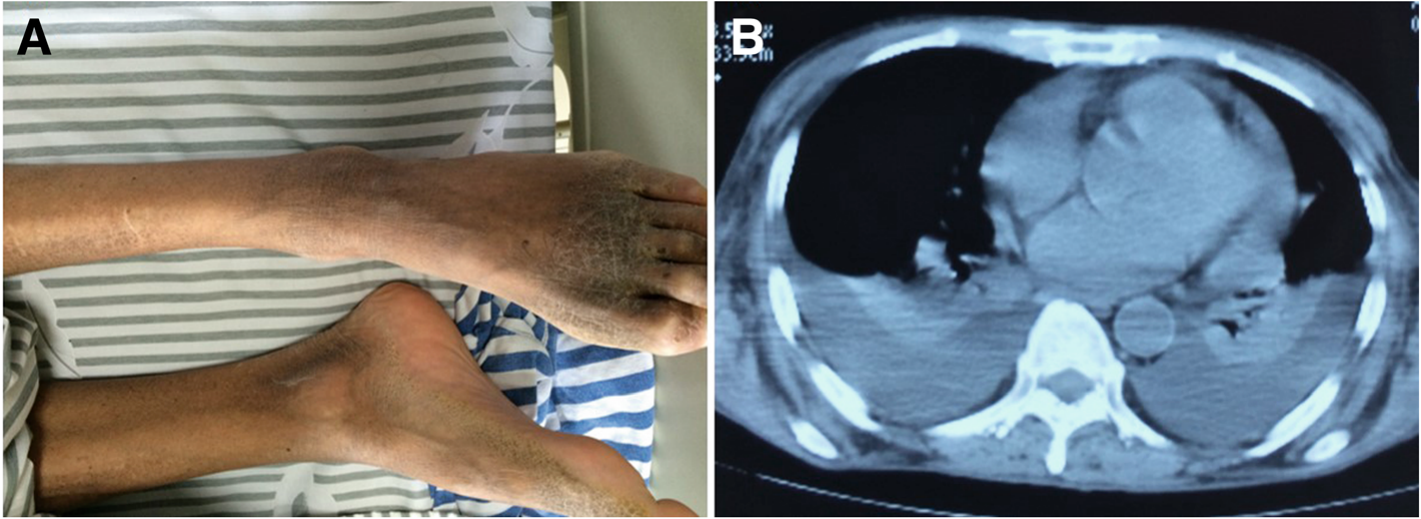
The clinical presentation of POEMS. The pigmentation with skin thickening on feet (**a**). The thoracic and abdominal CT showing splenomegaly(**b**)

**Fig. 3:**
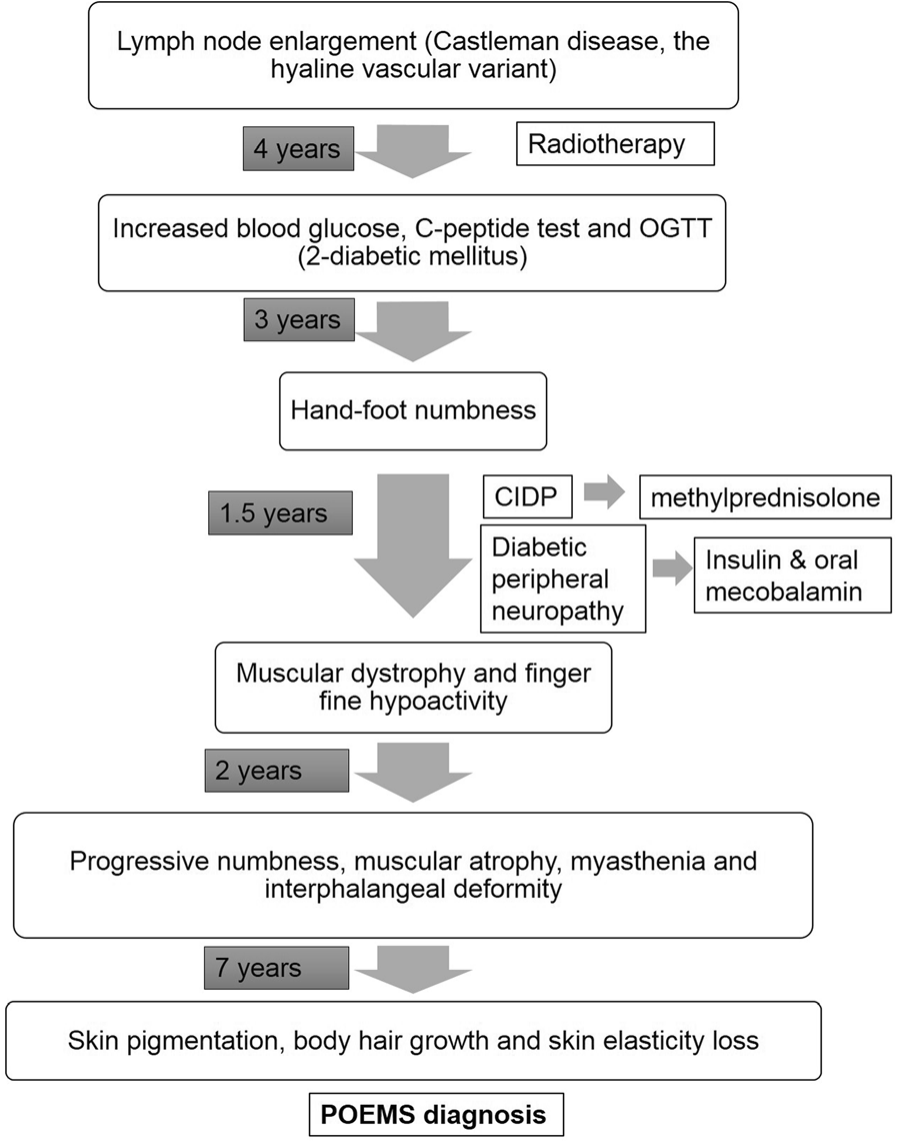
The schematic timeline of POEMS syndrome and accompanied treatments

The physical examination revealed that skin was thickened with hyperpigmentation over the toes (Fig. 2a). Other abnormal neurological examinations include the limb hypoesthesia, static tremor of hands, tendon hyporeflexia and positive Romberg sign. The thorax and abdominal CT scanning showed interstitial pneumonia and nodules located in right lobe, a small amount of pericardial effusion, hemorrhagic cyst on the pole of right kidney and splenomegaly (Fig. 2b). The pituitary gland MRI showed the shrunk size of pituitary gland concurrent with partial empty-sella and nasosinusitis. The ultrasonography excluded lesions of adrenal glands but discovered bilateral thoracic effusion post CT scanning. The electromyography test was repeated with severe to complete neuro-electrophysiological damage in bilateral median, ulnar, peroneal and tibial nerves and movement loss of tibialis anterior muscle and abductor muscle of right little finger. Laboratory investigations revealed a platelet count of 454 × 10^12^/L (normal, 125~ 350 × 10^9^/L), triglycerides level of 1.8 mmol/L (normal, 0.5~ 1.7 g/L), albumin of 20 g/L (normal, 35~ 50 g/L), adrenocorticotropic hormone (ACTH) level of 322.0 pg/ml which fell to 227.0 pg/ml (normal, 0~ 46 pg/ml) after one week. Her CRP level was 17.7 mg/L but rose to 21.3 mg/L (normal, 0~ 3.0 mg/L) within 2 weeks. The blood cortisol level in the morning was 220.3 nmol/l (normal, 138~ 690 nmol/L) within one week. The coagulation function showed PT-SEC of 15.6 s (normal, 10.0~ 14.0 s), PT% of 49.4% (normal, 70.0%~ 130.0%) and APTT-SEC of 44.2 s (normal, 20.0~ 40.0 s). The serum immunoglobin test indicated moderately increased IgG level of 11.9 g/L (normal, 7.6~ 11.6 g/L) and decreased complement C3 level of 0.5 g/L (normal, 0.8~ 1.4 g/L). Viral serology (EBV and HSV not included), urine free cortisol, tuberculosis antibody, sex hormone test, thyroid hormone and tumor biomarkers were undertaken and came back with normal value. BM cytology revealed significant hypercellularity and thrombocythemia. Genetic tests from BM biopsy showed negative expression of JAK-2 gene and BCR/ABL gene fusion, therefore the primary thrombocytosis was diagnosed instead of multiple myeloma. Abnormal hyperimmunoglobulinia appeared as minimal IgG-lambda monoclonal protein variant (Fig. 3). The clinical presentation and investigations consisted with features of POEMS syndrome. The patient was managed with daily oral prednisone of 20 mg and azathioprine of 150 mg for 2 weeks. The numbness was apparently relieved with improved performance status. Thus, thalidomide with oral dose of 50 mg daily was taken as maintenance therapy. Clinical monitor of response and toxicity was evaluated periodically within the next 2 years and the patient rejected medical treatment and died of hypoalbuminemia, electrolyte disturbance and pneumonia.

## Discussion and conclusions

POEMS syndrome is a rare paraneoplastic syndrome compromising polyneuropathy, organomegaly, endocrinopathy, monoclonal protein and skin changes [2]. The diagnosis is easily missed as it is generally established on a group of clinical onset and laboratory features. The mandatory criteria of POEMS diagnosis include polyneuropathy (typical demyelinating) and monoclonal plasma cell-proliferative disorder (almost always λ). CD (separate of POEMS syndrome variant), sclerotic bone lesions, vascular endothelial growth factor elevation constitute other major but not obligatory criteria. Other important features known as minor criteria are defined as organomegaly, extravascular volume overload, endocrinopathy, skin changes, papilledema and thrombocytosis or polycythemia [3]. Only those with two key components of the mandatory criteria could be diagnosed as standard POMES syndrome. Otherwise, the POEMS syndrome variant should be defined in case of complicated CD.

Our patient was initially diagnosed with Castleman disease without any clinical evidence of POEMS syndrome. The following radiotherapy showed good clinical response. After 4 years post radiotherapy, the first onset of manifestation was dysglycemia. The polyneuropathy in the form of limb numbness emerged in an interval of 2 years. The typical Castleman disease variant of POEMS syndrome has no clonal PCD and typically little to no peripheral neuropathy, therefore Castleman’s disease variant of POEMS syndrome could not be defined in our patient. Although he did not respond to the standard CIDP therapy and the alternative diabetic neuropathy standard therapy, the potential POEMS syndrome was not considered and obligatory laboratory tests including monoclonal protein (especially λ) test for its early diagnosis were therefore missed. The subsequent presence of skin pigmentation, splenomegaly and progressive polyneuropathy then elicitedthe clue of POEMS syndrome, which was confirmed by appearance of monoclonal protein(λ) (Fig. 3).

The incidence of POEMS in CD cases has not been accurately reported. Several published cases of CD with interesting features are likely cases of POEMS. In 30 POEMS syndrome cases, 19 biopsied lymph nodes showed Castleman disease of angiofollicular hyperplasia type in total of 32 samples [4]. In another report, 25 of 43 biopsied lymph nodes were pathological of Castleman disease, 84% of which was diagnostic of hyaline vascular type [5]. The concurrent cavity effusion and co-existing Castleman disease predicted poor prognostic and short survival, reflected by two-year survival interval post initiated therapy in this case [2, 5]. The pathogenesis of this syndrome is currently unknown, in which vascular epithelial growth factor (VEGF) might play a role as its remarkable elevation in most POEMS syndrome patients [6]. Although the serum and plasma level of VEGF correlated with activity of this disease [7–9], the use of anti-VEGF antibody remains controversial. Due to its rarity and heterogeneity, randomized clinical trials are hardly published to establish the consensus management, therefore, the treatment is majorly based on case reports and series. The successful managements aim to the inhibition of clonal plasma cell disorder instead of targeting VEGF pathway. In the case of localized plasma cell disease with sole bone lesion and no bone marrow infiltration, radiation is recommended. In condition of bone marrow dissemination, systemic chemotherapy is considered as the priority. Lenalidomide or thalidomide combined with dexamethasone has also exhibited clinical benefit on VEGF, peripheral neuropathy and extravascular volume overload [3], especially for patients with poor PS or opposed to chemotherapy. In consideration of poor ECOG (score 3) and exclusive bone marrow dissemination, our patient was finally managed with prednisone, azathioprine and sequential thalidomide as maintenance therapy with good clinical response, in line with previous studies. Although azathioprine is not considered as the mainstay of conventional systemic therapy in POEMS syndrome, its combination with corticosteroids showed promise with managed toxicity. Its benefit against peripheral polyneuropathy needs to be weighted in the future clinical trials.

In conclusion, the diagnosis of POEMS was based on a cluster of clinical manifestations and laboratory investigations. Especially for patients with CD history, the presence of polyneuropathy requires clinicians to distinguish from POEMS syndrome variant of CD to standard POEMS syndrome, therefore a detailed history collection and physical examination in addition to essential laboratory tests (bone marrow biopsy, VEGF level test and monoclonal protein) are necessary to rule in the diagnosis. The mainstays of POEMS syndrome therapy mainly target to plasma cell disorder or local lesions, dependent on the status of bone marrow infiltration. In our case, the long-term transition of treated CD to POEMS syndrome might give a clue to elaborating the pathogenesis of this disease in the future.

## Abbreviations

ACTH: Adrenocorticotropic hormone
BM: Bone marrow
CD: Castleman disease
CIDP: Chronic inflammatory demyelinating polyneuropathy
CSF: The cerebrospinal fluid
OGTT: Oral glucose tolerance test
VEGF: Vascular epithelial growth factor
